# Growth Arrest of Staphylococcus aureus Induces Daptomycin Tolerance via Cell Wall Remodelling

**DOI:** 10.1128/mbio.03558-22

**Published:** 2023-02-01

**Authors:** Elizabeth V. K. Ledger, Andrew M. Edwards

**Affiliations:** a MRC Centre for Molecular Bacteriology and Infection, Imperial College London, London, United Kingdom; New York University Grossman School of Medicine

**Keywords:** *Staphylococcus aureus*, daptomycin, antibiotic tolerance, peptidoglycan, growth arrest, MRSA

## Abstract

Almost all bactericidal drugs require bacterial replication and/or metabolic activity for their killing activity. When these processes are inhibited by bacteriostatic antibiotics, bacterial killing is significantly reduced. One notable exception is the lipopeptide antibiotic daptomycin, which has been reported to efficiently kill growth-arrested bacteria. However, these studies employed only short periods of growth arrest (<1 h), which may not fully represent the duration of growth arrest that can occur *in vivo*. We found that a growth inhibitory concentration of the protein synthesis inhibitor tetracycline led to a time-dependent induction of daptomycin tolerance in S. aureus, with an approximately 100,000-fold increase in survival after 16 h of growth arrest, relative to exponential-phase bacteria. Daptomycin tolerance required glucose and was associated with increased production of the cell wall polymers peptidoglycan and wall-teichoic acids. However, while the accumulation of peptidoglycan was required for daptomycin tolerance, only a low abundance of wall teichoic acid was necessary. Therefore, whereas tolerance to most antibiotics occurs passively due to a lack of metabolic activity and/or replication, daptomycin tolerance arises via active cell wall remodelling.

## INTRODUCTION

All antibiotics disrupt essential processes or structures in bacteria (1, 2). Whether this disruption leads to bacterial killing or growth inhibition is a function of the antibiotic class, the concentration, and the physiological state of the cell (1–11). Understanding the factors that modulate antibiotic activity is important because the inability of bactericidal antibiotics to kill bacteria has been linked to treatment failure and to the emergence of antibiotic resistance (12–17). This is particularly important in the case of Staphylococcus aureus, since this pathogen causes several refractory infections, including bacteremia, infective endocarditis, osteomyelitis, and implant infections (12–14, 18).

It is well-established that the growth arrest of S. aureus, for example, via exposure to a growth inhibitory concentration of a bacteriostatic antibiotic, significantly increases bacterial survival during a subsequent exposure to most bactericidal antibiotics (3, 8–10).

However, the reasons for this are subject to debate because, in addition to blocking replication, bacteriostatic antibiotics also compromise respiration and thereby compromise metabolic pathways (10). Since a lack of metabolic activity results in a lack of replication, it has been challenging to determine whether killing by bactericidal antibiotics requires active metabolism or replication (or both) to occur. However, a recent study provided evidence that metabolic activity is more important than replication for the killing of the human and animal pathogen S. aureus by several different antibiotics, and an earlier report linked a lack of cellular ATP in this bacterium to antibiotic tolerance (8, 11). Since the physiological states of bacteria at infection sites are likely to be different from those in laboratory media, understanding the host and bacterial factors that modulate antibiotic susceptibility may lead to better treatment outcomes (12, 13).

One of the few antibiotics that has been reported to efficiently kill growth-arrested S. aureus is the cyclic lipopeptide daptomycin, which is used to treat infections that are caused by drug-resistant Gram-positive pathogens, such as methicillin resistant S. aureus (MRSA) (19–21). Daptomycin targets phosphatidylglycerol (PG) and lipid II in the membrane, where it forms oligomeric complexes, which lead to membrane depolarization and permeabilization (19, 22–24). The binding of daptomycin to lipid II also disrupts cell wall synthesis, which is further compromised by the mislocalization of enzymes involved in peptidoglycan synthesis (19, 24–26).

The ability of daptomycin to kill nonreplicating bacteria has been demonstrated using cells that have been growth arrested (growth is blocked using bacteriostatic antibiotics or membrane depolarising agents) or cells that are not replicating due to cold temperature or entry into stationary-phase, which are conditions that almost completely block killing by other bactericidal antibiotics (10, 20, 21). However, while daptomycin maintains bactericidal activity against nonreplicating bacteria, these and other studies clearly showed that the degree and rate of killing of nonreplicating bacteria was reduced, compared to replicating bacteria (10, 20, 21, 27–29). Furthermore, these studies typically employed short periods of growth arrest (<1 h) and so we hypothesized that extended periods of growth inhibition, as can occur during persistent or recurrent infections, would further reduce daptomycin susceptibility.

The reason for the reduced susceptibility of nonreplicating S. aureus to killing by daptomycin is unknown (11), but it may have important clinical implications, as the potent activity of this antibiotic *in vitro* is not always replicated *in vivo*. In a retrospective analysis, >25% of patients with staphylococcal bacteremia who were given daptomycin monotherapy died (30), whereas other studies have reported treatment failure rates of 11 to 25% (31–33), demonstrating that there is a pressing need to improve daptomycin-based treatment approaches.

Therefore, the aims of this work were to determine the degree to which growth arrest confers daptomycin tolerance, to determine the mechanism responsible, and to identify ways to prevent tolerance and enhance daptomycin treatment efficacy.

## RESULTS

### Antibiotic-mediated growth arrest induces daptomycin tolerance in a time- and nutrient-dependent manner.

To test whether the growth arrest of S. aureus led to daptomycin tolerance, we grew bacteria to mid-exponential phase in tryptic soy broth (TSB) and then exposed them directly to daptomycin, or we incubated cells with a bacteriostatic concentration of the protein synthesis inhibitor tetracycline for 16 h to arrest growth before exposure to daptomycin. Tetracycline is the first member of the tetracycline class of antibiotics, and it is used to treat staphylococcal skin and soft tissue infections (34).

**FIG 1:**
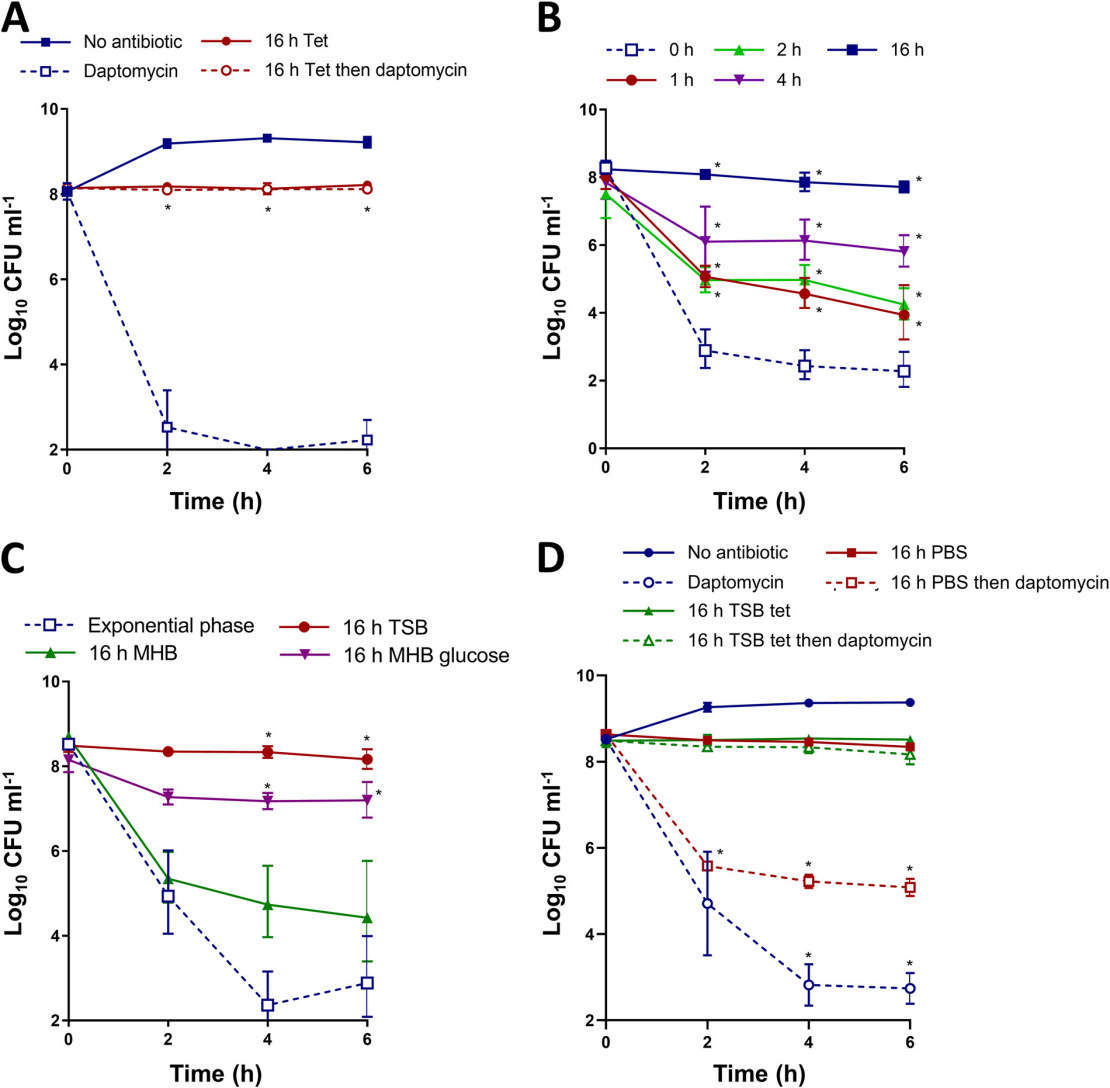
Tetracycline-mediated growth arrest induces daptomycin tolerance in a time- and nutrient-dependent manner. (A) S. aureus USA300 LAC* was grown to mid-exponential phase or grown to mid-exponential phase and then incubated for 16 h with 1.25 μg mL^−1^ tetracycline. Then, the log_10_ CFU mL^−1^ were determined throughout a 6 h exposure to 20 μg mL^−1^ daptomycin. (B) S. aureus USA300 LAC* was grown to mid-exponential phase and incubated for the indicated lengths of time with 1.25 μg mL^−1^ tetracycline before the log_10_ CFU mL^−1^ were determined throughout a 6 h exposure to 20 μg mL^−1^ daptomycin. (C) S. aureus USA300 LAC* was grown to mid-exponential phase or incubated for 16 h with 1.25 μg mL^−1^ tetracycline in TSB, MHB, or MHB supplemented with 2.5 g L^−1^ glucose before being exposed to 20 μg mL^−1^ daptomycin in TSB for 6 h. Then, the log_10_ CFU mL^−1^ were determined. (D) Log_10_ CFU mL^−1^ of mid-exponential-phase cells or cultures incubated in TSB supplemented with 1.25 μg mL^−1^ tetracycline or in PBS for 16 h, during a 6 h exposure to 20 μg mL^−1^ daptomycin in TSB. The data represent the geometric mean ± the geometric standard deviation of three independent experiments. The data in panel A were analyzed via a two-way ANOVA with Sidak’s *post hoc* test (*, *P* < 0.05; daptomycin-treated tetracycline-arrested versus exponential-phase). The data in panel B were analyzed via a two-way ANOVA with Tukey’s *post hoc* test (*, *P* < 0.05; nonarrested versus tetracycline-arrested). The data in panel C were analyzed via a two-way ANOVA with Dunnett’s *post hoc* test (*, *P* < 0.05; exponential-phase versus tetracycline-arrested). The data in panel D were analyzed via a two-way ANOVA with Dunnett’s *post hoc* test (*, *P* < 0.05; tetracycline-arrested versus nonarrested/PBS-arrested). Tet, tetracycline.

As expected, exponential phase S. aureus was highly susceptible to daptomycin, with an approximately 6 log reduction in CFU counts after 6 h of antibiotic exposure (Fig. 1A). In contrast, growth arrest with tetracycline completely prevented daptomycin killing (Fig. 1A).

To confirm that the survival of growth-arrested bacteria in TSB was not due to the selection of daptomycin-resistant mutants, we sampled three randomly selected colonies from exponential-phase bacteria with no exposure to daptomycin, bacteria that were growth arrested by tetracycline but not exposed to daptomycin, bacteria exposed to daptomycin only, and bacteria that were growth arrested by tetracycline and exposed to daptomycin. Under all conditions tested, the median daptomycin MIC was 0.5 μg mL^−1^, demonstrating that the survival of the growth arrested bacteria that were exposed to daptomycin was not due to selection for resistance (Fig. S1).

10.1128/mbio.03558-22.1 FIG S1 The survival of tetracycline growth-arrested bacteria exposed to daptomycin is not due to the selection for resistance to the lipopeptide antibiotic. S. aureus USA300 was grown to the mid-exponential phase (Exp) or growth-arrested for 16 h with 1.25 μg mL^−1^ tetracycline (Tet) before being challenged, or not, with 20 μg mL^−1^ daptomycin (dapt) for 6 h and plated onto TSA. The daptomycin MICs of three individual colonies from each of these conditions were measured. The data represent the median MIC of these three colonies. Download FIG S1, TIF file, 0.8 MB. Copyright © 2023 Ledger and Edwards. 2023 Ledger and Edwards. https://creativecommons.org/licenses/by/4.0/ This content is distributed under the terms of the Creative Commons Attribution 4.0 International license.

Next, we tested whether higher concentrations of daptomycin could overcome the growth arrest-induced tolerance phenotype. This revealed that growth-arrested bacteria remained tolerant, even at 160 μg mL^−1^ daptomycin, a concentration that killed exponential-phase bacteria to below the limit of detection within 2 h (Fig. S2A and B). We also found that the concentration of tetracycline used did not affect tolerance, provided it arrested growth (Fig. S2C).

10.1128/mbio.03558-22.2 FIG S2 Dose-dependent effects on the killing and tolerance of daptomycin and tetracycline. (A) Log_10_ CFU mL^−1^ of mid-exponential-phase S. aureus USA300 during a 6 h exposure to a range of concentrations of daptomycin. (B) Log_10_ CFU mL^−1^ of S. aureus USA300 which had been growth-arrested for 16 h with 1.25 μg mL^−1^ tetracycline during a 6 h exposure to a range of concentrations of daptomycin. (C) Log_10_ CFU mL^−1^ of S. aureus USA300 which had been growth-arrested for 16 h with a range of concentrations of tetracycline during a 6 h exposure to 20 μg mL^−1^ daptomycin. The data in panels A to C represent the geometric mean ± the geometric standard deviation of three independent replicates, and they were analyzed via a two-way ANOVA with Dunnett’s *post hoc* test (*, *P* < 0.05; survival at each time-point versus 0 h). Download FIG S2, TIF file, 1.2 MB. Copyright © 2023 Ledger and Edwards. 2023 Ledger and Edwards. https://creativecommons.org/licenses/by/4.0/ This content is distributed under the terms of the Creative Commons Attribution 4.0 International license.

Since the 16 h of growth arrest used here was longer than that used in previous studies (10, 20, 21), we next examined how the duration of growth inhibition affected daptomycin tolerance. In agreement with previous work (10, 20, 21), a short period of growth inhibition (1 or 2 h) reduced daptomycin killing by approximately 100-fold, relative to growing bacteria (Fig. 1B). Cells that were growth-arrested for 4 h showed a further reduction in susceptibility to daptomycin, with 4 log higher survival, relative to growing S. aureus cells, whereas 16 h of incubation in tetracycline again conferred complete protection from daptomycin-mediated killing (Fig. 1B).

Since the degree of daptomycin tolerance was time-dependent, we hypothesized that an active process was required for the loss of antibiotic susceptibility. Therefore, we investigated the nutritional requirements for tolerance by arresting growth for 16 h with tetracycline in either TSB or Müller-Hinton broth (MHB), which are media with different nutrient compositions.

While 16 h of growth arrest in TSB completely blocked killing by daptomycin, this was not the case with MHB, with a >1,000-fold reduction in CFU being observed after 6 h of exposure to the lipopeptide antibiotic (Fig. 1C). One of the differences between TSB and MHB is that the former contains glucose (2.5 g L^−1^), and the supplementation of MHB with this sugar demonstrated that it was crucial for the induction of tolerance (Fig. 1C). To test the requirements for nutrients and to determine whether daptomycin tolerance was due to a lack of growth or metabolic activity, we starved bacteria for 16 h in phosphate-buffered saline (PBS), which does not permit replication (Fig. 1D). Nutrient limitation failed to provide the high level of daptomycin tolerance seen in TSB, with a 3 log reduction of CFU counts being observed within 2 h of daptomycin exposure (Fig. 1D). We then examined which sugars are needed for tolerance. Since previous work has shown that growth arrest with antibiotics such as tetracycline suppresses respiration, we examined a range of sugars shown to support S. aureus growth, or not, under anaerobic conditions (35). Fermentable carbohydrates that have previously been reported to support full S. aureus growth under anaerobic conditions (glucose, sucrose) could support the induction of tolerance due to growth arrest, whereas sugars that were not found to be good growth substrates in the absence of oxygen (galactose, lactose, sorbitol) did not promote tolerance above the level seen for MHB only (Fig. S3) (35). We also found that the trisaccharide raffinose did not support high-level tolerance induction (Fig. S3). To test whether oxygen was needed to induce tolerance, we growth arrested S. aureus in MHB with glucose and incubated it at 37°C without shaking for 16 h. In agreement with previous work showing that tetracycline suppresses respiration, aeration was not required for the induction of tolerance in MHB containing glucose (Fig. S3). Finally, we found additional evidence that metabolic processes are needed for the induction of tolerance, since bacteria that were growth arrested with tetracycline in MHB with glucose at 4°C for 16 h were extremely susceptible to killing by daptomycin (Fig. S3).

10.1128/mbio.03558-22.3 FIG S3 Growth arrest-induced daptomycin tolerance requires fermentable sugars but not aeration. Mid-exponential phase S. aureus USA300 cells were growth-arrested with 1.25 μg mL^−1^ tetracycline for 16 h at 37°C with shaking in MHB or in MHB supplemented with 2.5 g L^−1^ (13.9 mM) glucose or equimolar concentrations of galactose, sorbitol, sucrose, raffinose, or lactose, or with 2.5 g L^−1^ glucose under static conditions, or at 4°C under static conditions, before being exposed to 20 μg mL^−1^ daptomycin for 6 h. Survival was determined before and after daptomycin exposure via CFU counts. The data represent the geometric mean ± the geometric standard deviation of three independent biological replicates, and they were analyzed via a two-way ANOVA with Sidak’s *post hoc* test (*, *P* < 0.05; survival at 6 h versus 0 h). Download FIG S3, TIF file, 1.6 MB. Copyright © 2023 Ledger and Edwards. 2023 Ledger and Edwards. https://creativecommons.org/licenses/by/4.0/ This content is distributed under the terms of the Creative Commons Attribution 4.0 International license.

10.1128/mbio.03558-22.4 FIG S4 The DiSC_3_(5) dye binds equally well to exponential-phase and growth-arrested bacteria after depolarization with nigericin. S. aureus USA300 cells were grown to the mid-exponential phase or were incubated for 16 h with 1.25 μg mL^−1^ tetracycline before being exposed to 500 μg mL^−1^ nigericin for 1 h. The membrane potential was measured with DiSC_3_(5). The data represent the mean ± the standard deviation of three independent experiments, and they were analyzed via a paired *t* test (*P* > 0.05). n.s. nonsignificant. Download FIG S4, TIF file, 0.7 MB. Copyright © 2023 Ledger and Edwards. 2023 Ledger and Edwards. https://creativecommons.org/licenses/by/4.0/ This content is distributed under the terms of the Creative Commons Attribution 4.0 International license.

Together, these data showed that growth arrest led to high levels of daptomycin tolerance via a time-dependent and nutrient-dependent process, in contrast to the tolerance of other bactericidal antibiotics, which occurs after short periods of growth arrest or a lack of metabolic activity (7, 9, 11).

### Daptomycin tolerance requires the synthesis of cell surface components.

Next, we aimed to determine which biosynthetic pathways were required for daptomycin tolerance by investigating the effects of growth arrest by various classes of antibiotics. To do this, we incubated exponential phase cultures of S. aureus for 16 h with growth inhibitory concentrations of various classes of antibiotics that each have distinct targets, including chloramphenicol (inhibitor of protein synthesis), ciprofloxacin (fluoroquinolone, inhibitor of DNA replication), cephalexin (β-lactam, inhibitor of cell wall synthesis), tunicamycin (inhibitor of wall teichoic acid [WTA] synthesis), or AFN-1252 (inhibitor of fatty acid synthesis), and we then challenged the cultures with daptomycin.

**FIG 2:**
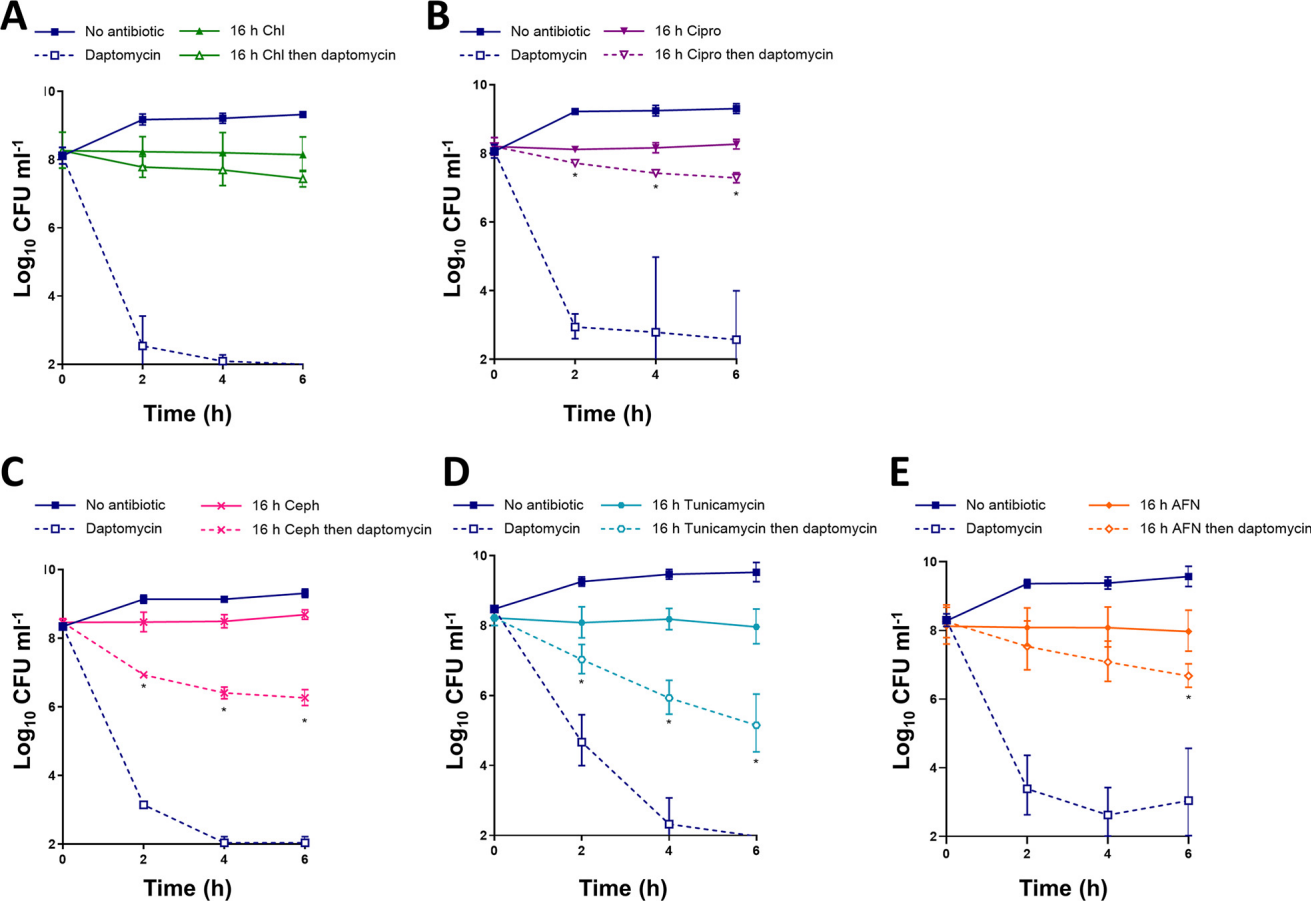
Antibiotics with different mechanisms of action induce tolerance to different degrees. S. aureus USA300 LAC* were grown to mid-exponential phase or were incubated for 16 h with (A) 20 μg mL^−1^ chloramphenicol, (B) 160 μg mL^−1^ ciprofloxacin, (C) 32 μg mL^−1^ cephalexin, (D) 0.5 μg mL^−1^ tunicamycin, or (E) 0.15 μg mL^−1^ AFN-1252 before the log_10_ CFU mL^−1^ were determined throughout a 6 h exposure to 20 μg mL^−1^ daptomycin. The data represent the geometric mean ± the geometric standard deviation of three independent experiments. The data were analyzed via a two-way ANOVA with Sidak’s *post hoc* test (*, *P* < 0.05; daptomycin-treated antibiotic-arrested versus daptomycin-treated exponential-phase). Chl, chloramphenicol; Cipro, ciprofloxacin; Ceph, cephalexin; AFN, AFN-1252.

Growth arrest with each of the antibiotics led to various degrees of daptomycin tolerance (Fig. 2A–E). Similar to tetracycline, the inhibition of protein synthesis with chloramphenicol led to high levels of tolerance, with daptomycin killing fewer than 1 log of the S. aureus CFU (Fig. 2A). Growth arrest with ciprofloxacin also led to a high degree of tolerance (Fig. 2B). However, growth arrest with antibiotics that inhibited the synthesis of components of the cell surface (peptidoglycan, WTA, or fatty acids) resulted in lower levels of daptomycin tolerance (Fig. 2C, D, E). This was especially pronounced with cephalexin and tunicamycin, with which daptomycin caused 2 to 3 log reductions of CFU within 6 h (Fig. 2C, D).

Combined, these data showed that growth arrest by different classes of antibiotics resulted in various degrees of daptomycin tolerance but that full tolerance required peptidoglycan, WTA, and fatty acid synthesis, suggesting that changes to the bacterial cell surface contributed to tolerance.

### Antibiotic-mediated growth arrest reduces daptomycin binding and membrane damage.

To understand how growth arrest-induced changes to the cell wall reduced daptomycin susceptibility, we next tested whether antibiotic-mediated growth arrest affected the ability of daptomycin to bind to its membrane target.

**FIG 3:**
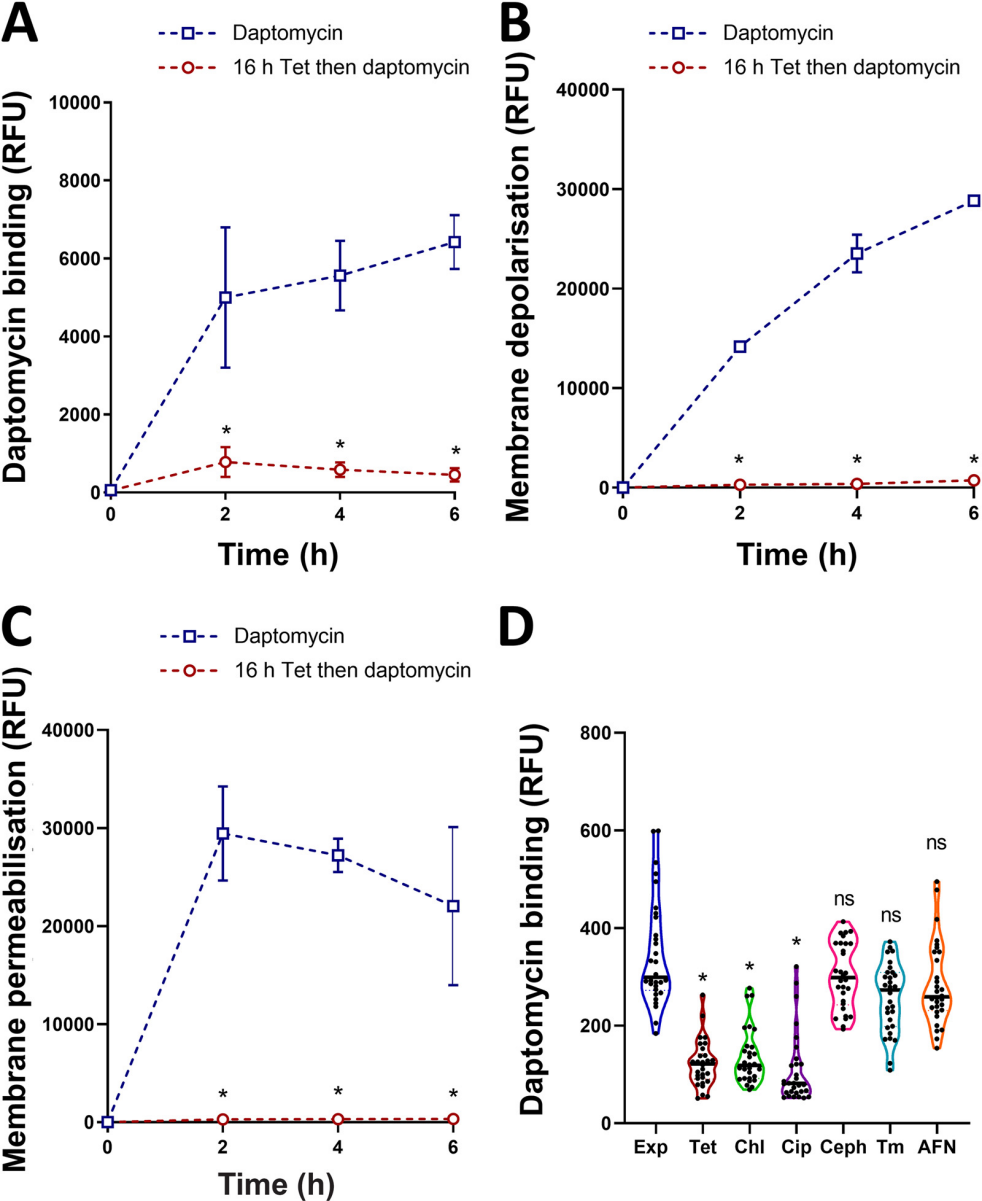
Antibiotic-mediated growth arrest reduces daptomycin binding and membrane damage. (A) S. aureus USA300 LAC* were grown to mid-exponential phase or were incubated for 16 h with 1.25 μg mL^−1^ tetracycline before being exposed to 80 μg mL^−1^ BoDipy-daptomycin. Then, the cell-associated fluorescence was determined. (B) DiSC_3_(5) and (C) propidium iodide fluorescence of mid-exponential or tetracycline-arrested cultures during a 6 h exposure to 20 μg mL^−1^ daptomycin. (D) BoDipy-daptomycin binding to exponential-phase and antibiotic-arrested cultures of S. aureus USA300 LAC*. The cultures were incubated with 80 μg mL^−1^ BoDipy-daptomycin for 2 h, washed, fixed, and analyzed via fluorescence microscopy. The fluorescence of 30 cells per condition was measured. The data in panels A to C represent the mean ± standard deviation of three independent experiments. The data in panel D represent individual cellular measurements, with the median indicated. The data in panels A to C were analyzed via a two-way ANOVA with Tukey’s *post hoc* test (*, *P* < 0.05; tetracycline-arrested versus exponential-phase). The data in panel D were analyzed via a Kruskal-Wallis test with Dunn’s *post hoc* test (*, *P* < 0.05; Exp versus antibiotic-arrested). Exp, exponential phase; Tet, tetracycline; Chl, chloramphenicol; Cip, ciprofloxacin; Ceph, cephalexin; Tm, tunicamycin; AFN, AFN-1252.

To do this, we measured the attachment of fluorescent BoDipy-daptomycin to mid-exponential phase or tetracycline-arrested cultures over a 6 h period, with aliquots being taken every 2 h to determine the kinetics of the antibiotic binding. In line with the bacterial survival data, the exponential phase cells were bound by high levels of daptomycin within 2 h, whereas the growth-arrested cells had up to 15-fold lower levels of bound daptomycin (Fig. 3A). This finding is similar to those in previous reports that correlated the increased binding of BoDipy-daptomycin with the decreased survival of S. aureus or Enterococcus faecalis (36–39).

Next, we measured membrane depolarization and permeability using the fluorescent dyes DiSC_3_(5) and propidium iodide, respectively. As expected from previous work (40), the membranes of exponential-phase S. aureus were rapidly depolarized and permeabilized on exposure to daptomycin (Fig. 3B, C). In contrast, no membrane disruption was detected by either assay in the tetracycline growth-arrested cultures (Fig. 3B, C). To ensure that the DiSC_3_(5) dye could bind the membranes of the growth-arrested bacteria, we used nigericin as a positive control and showed the equal staining of the depolarized membranes of exponential-phase and growth-arrested cells (Fig. S4).

Finally, we investigated the effect of growth arrest by other classes of antibiotics on daptomycin binding. As described above, growth arrest with tetracycline significantly reduced daptomycin binding (Fig. 3D). In agreement with the data from bacterial survival assays (Fig. 2A, B, C, D, E), chloramphenicol and ciprofloxacin also significantly reduced daptomycin binding, compared to the exponential-phase cultures, whereas BoDipy-daptomycin bound as well to cells that were growth-arrested with cephalexin, tunicamycin, or AFN-1252 as it did to the exponential-phase cultures (Fig. 3D).

Taken together, these data demonstrated that the daptomycin tolerance of growth-arrested bacteria was due to reduced binding of the lipopeptide to its membrane targets, and this likely resulted from changes to the cell envelope.

### Daptomycin tolerance requires changes to the cell wall but not the membrane.

The next objective was to determine which components of the cell surface were required for daptomycin tolerance. As changes to both the cell wall and the membrane components of S. aureus have previously been associated with reduced daptomycin susceptibility (19, 40–48), we tested each in turn, starting with membrane phospholipid synthesis.

**FIG 4:**
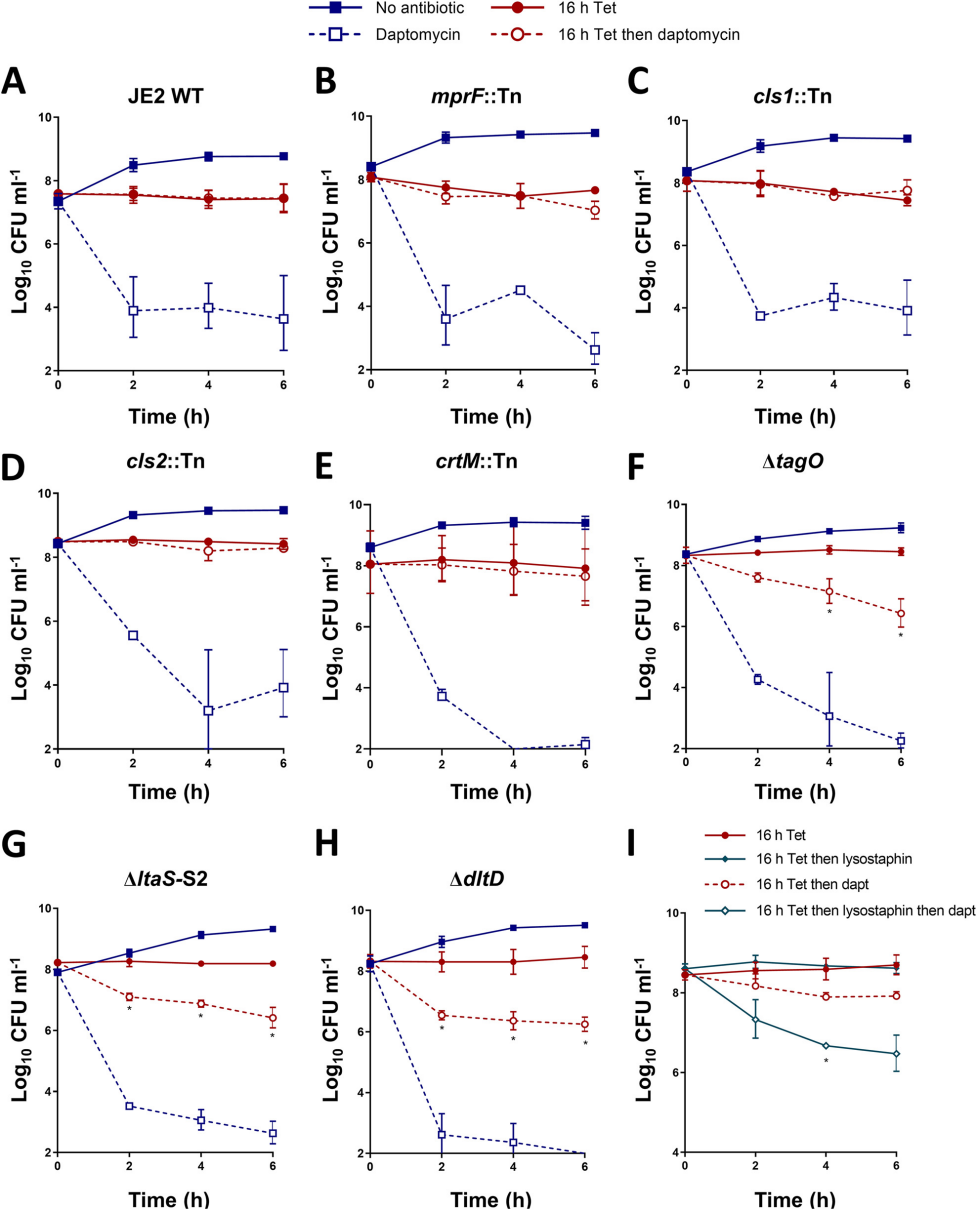
Daptomycin tolerance requires changes in the cell wall and not in the cell membrane. Log_10_ CFU mL^−1^ of exponential-phase and tetracycline-arrested cultures of (A) JE2 WT, (B) JE2 *mprF*::Tn, (C) JE2 *cls1*::Tn, (D) JE2 *cls2*::Tn, (E) JE2 *crtM*::Tn, (F) LAC* Δ*tagO*, (G) LAC* Δ*ltaS*-S2, or (H) LAC* Δ*dltD* during a 6 h exposure to 20 μg mL^−1^ daptomycin. (I) Log_10_ CFU mL^−1^ of tetracycline-arrested cultures of S. aureus LAC*, after the partial digestion of the cell wall with lysostaphin, or not, and during a 6 h exposure to 20 μg mL^−1^ daptomycin. The data represent the geometric mean ± the geometric standard deviation of three independent experiments. The data in panels A to H were analyzed via a two-way ANOVA with Sidak’s *post hoc* test (*, *P* < 0.05; tetracycline-arrested versus tetracycline-arrested + daptomycin). The data in panel I were analyzed via a two-way ANOVA with Dunnett’s *post hoc* test (*, *P* < 0.05; lysostaphin treated versus untreated). Tet, tetracycline; Dapt, daptomycin.

The main phospholipid species in the S. aureus membrane are phosphatidylglycerol (PG), lysyl-phosphatidylglycerol (LPG), and cardiolipin (CL) (43, 45). As PG is essential, we investigated whether the synthesis of either LPG (by MprF) or CL (by either Cls1 or Cls2) were required for tolerance, using mutants defective for these proteins from the NARSA library, which was generated in the USA300 LAC JE2 background. Therefore, we confirmed that tetracycline induced daptomycin tolerance in the JE2 WT strain (Fig. 4A) before testing the phenotype of each of the mutants. Similar to the WT strain, growth arrest conferred daptomycin tolerance in each of the three phospholipid synthase mutants, with no significant killing being observed in any of the strains (Fig. 4B, C, D).

During our assays, we observed that the growth-arrested S. aureus were much more strongly pigmented than were the exponential-phase cultures. This pigment, staphyloxanthin, is a major factor influencing membrane fluidity, and it has been implicated in reducing daptomycin susceptibility (48). Therefore, we next tested a mutant defective in pigment synthesis, *crtM*::Tn. However, despite lacking staphyloxanthin, this mutant also showed high levels of daptomycin tolerance after being incubated with tetracycline, confirming that the synthesis of this pigment did not contribute to bacterial survival (Fig. 4E). Therefore, we found no evidence that daptomycin tolerance was due to changes in membrane phospholipid composition or staphyloxanthin content.

Teichoic acids are major components of the cell envelope, and they can be either covalently bound to the cell wall (WTA) or anchored to the membrane (lipoteichoic acid, LTA) (49). These polymers are both modified with d-alanine groups by the products of the *dltABCD* operon, which reduces the net negative charge of the surface (49). To test whether either of these polymers were required for tolerance, we examined mutants lacking WTA or LTA (Δ*tagO* or Δ*ltaS*-S2, respectively) or lacking the d-alanine modification (Δ*dltD*). These assays showed that the survival of each mutant during daptomycin exposure was approximately 2 log lower than that of the WT after 6 h, indicating that the presence of d-alanylated teichoic acids was required for full tolerance (Fig. 4F, G, H).

To further test whether tolerance required the cell wall, we arrested the growth of S. aureus with tetracycline to induce tolerance. Then, we partially digested peptidoglycan with lysostaphin and measured the daptomycin susceptibility. Lysostaphin treatment led to an approximately 2 log reduction in bacterial survival, compared to cells with an intact cell wall, confirming a key role for the cell wall in mediating daptomycin tolerance that was induced by growth arrest with tetracycline (Fig. 4I).

Taken together, the induction of daptomycin tolerance in growth-arrested cells was due to changes to the cell wall but not the membrane.

### Antibiotic-mediated growth arrest causes peptidoglycan and WTA accumulation.

Having established that the cell wall was required for growth arrest-induced daptomycin tolerance, we next aimed to determine the nature of the changes associated with tolerance.

**FIG 5:**
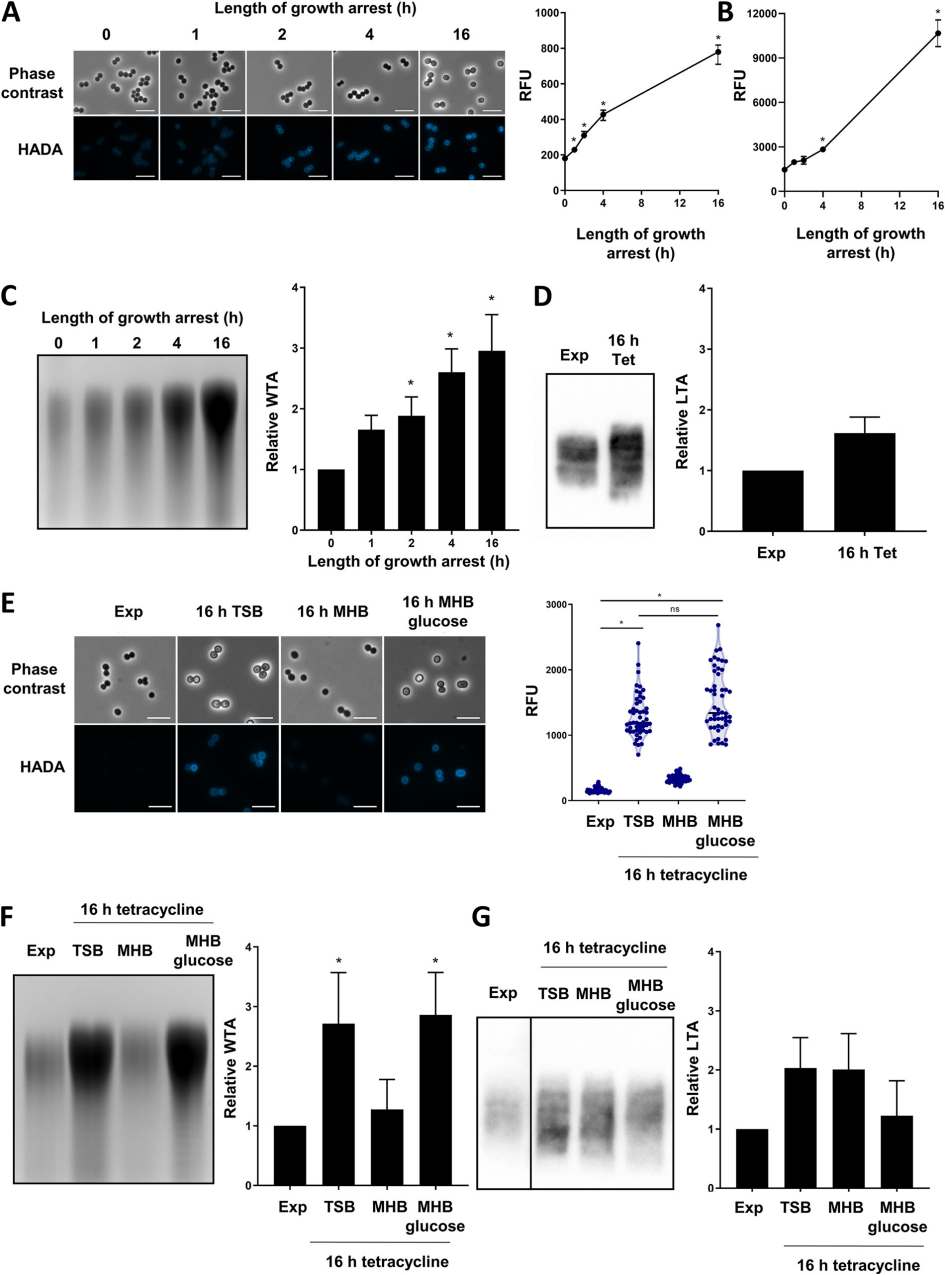
Growth arrest with tetracycline leads to increases in peptidoglycan and WTA. Exponential-phase S. aureus LAC* were growth-arrested with tetracycline for the indicated lengths of time in the presence of 25 μM HADA before being analyzed via (A) phase contrast and fluorescence microscopy, with the fluorescence of the cell surface of individual cells being quantified, and via (B) flow cytometry. Exponential phase S. aureus LAC* were growth-arrested with tetracycline for the indicated lengths of time before (C) WTA was extracted, analyzed via native PAGE, visualized with Alcian blue, and quantified, or (D) LTA was analyzed via Western blotting and quantified. The data are expressed as relative to the exponential-phase values. (E) Tetracycline-mediated growth arrest was carried out in TSB, MHB, or MHB supplemented with 2.5 g L^−1^ glucose for 16 h in the presence of 25 μM HADA before the cells were visualized via phase contrast and fluorescence microscopy and the fluorescence of the surfaces of individual cells were quantified. Tetracycline-mediated growth arrest was carried out in TSB, MHB, or MHB supplemented with 2.5 g L^−1^ glucose for 16 h before (F) WTA was extracted, analyzed via PAGE, and quantified with Alcian blue staining. (G) LTA was extracted, analyzed via Western blotting and quantified. The data are expressed as relative to the exponential-phase values. The data in panel A represent the median fluorescence ± 95% confidence intervals of 50 individual cells. The data in panels B, C, D, F, and G represent the mean ± the standard deviation of three independent experiments. The data in panel E represent the fluorescence values of individual cells, with the median indicated. WTA PAGE and LTA Western blots were performed three times, and representative images are shown. The data in panels A and B were analyzed via a one-way ANOVA with Dunnett’s *post hoc* test (*, *P* < 0.05; indicated time point versus 0 h). The data in panel C were analyzed via a one-way ANOVA with Tukey’s *post hoc* test (*, *P* < 0.05; tetracycline-arrested versus exponential-phase). The data in panel C were analyzed via a *t* test (no significant difference). The data in panel E were analyzed via a Kruskal-Wallis test with Dunn’s *post hoc* test (*, *P* < 0.05). The data in panels F and G were analyzed via a two-way ANOVA with Dunnett’s *post hoc* test (*, *P* < 0.05; tetracycline-arrested versus exponential-phase). No statistically significant differences were observed in panel G. Exp, exponential phase; Tet, tetracycline; TSB, tryptic soy broth; MHB, Müller-Hinton broth.

First, we looked at whether growth arrest led to increases in any of the three main components of the cell wall: peptidoglycan, WTA, and LTA. To test whether peptidoglycan was accumulating, we used HADA, a fluorescent d-amino acid that is incorporated into peptidoglycan as it is synthesized (50). The growth arrest was carried out in TSB supplemented with HADA for various lengths of time before the bacteria were imaged and the fluorescence was quantified via microscopy (Fig. 5A) and flow cytometry (Fig. 5B). This demonstrated that longer periods of growth arrest were associated with higher levels of HADA fluorescence, indicating that peptidoglycan was accumulating over time (Fig. 5A, B). In keeping with the changes to the cell envelope, the appearances of the cells varied over time when imaged via phase-contrast microscopy, with the bacteria becoming bigger and more opaque (Fig. 5A).

Next, we extracted and quantified WTA from the exponential-phase and growth-arrested cultures, and this demonstrated a time-dependent increase in the quantity of WTA, with three times more WTA being present after a 16 h growth arrest than was observed in the exponential-phase cultures (Fig. 5C). In contrast, the extraction and detection of LTA via Western blotting revealed no significant increase in the quantity of LTA in the growth-arrested cells, compared to those in the exponential phase (Fig. 5D). However, the migration of LTA from growth-arrested cells was different from that of growing bacteria, which may reflect differences in the LTA structure or membrane anchor (51).

Having identified that growth arrest led to time-dependent increases in both peptidoglycan and WTA, but not in LTA, we next investigated whether the increases in these polymers were nutrient-dependent. In line with the tolerance data (Fig. 1C), both peptidoglycan and WTA abundance increased when growth arrest occurred in TSB, whereas no significant accumulation of either polymer was observed after growth arrest in MHB (Fig. 5E, F). Furthermore, bacteria that were growth-arrested in the presence of glucose had a different appearance, compared to those growth-arrested without this sugar, which may reflect differences in the cell envelope. Indeed, growth arrest in MHB supplemented with glucose led to increased levels of both peptidoglycan and WTA (Fig. 5E, F). As confirmation that the increased LTA was not associated with growth arrest-induced daptomycin tolerance, we found no differences between the levels of LTA in cells arrested in TSB or MHB, and the supplementation of the MHB with glucose did not lead to increased LTA (Fig. 5G).

Taken together, tetracycline-mediated growth arrest led to time- and nutrient-dependent increases in peptidoglycan and WTA, but not in LTA.

### Daptomycin tolerance is due to the accumulation of peptidoglycan but not WTA.

The final objective was to determine whether the accumulation of either peptidoglycan or WTA that occurred during the growth arrest was responsible for tolerance.

**FIG 6:**
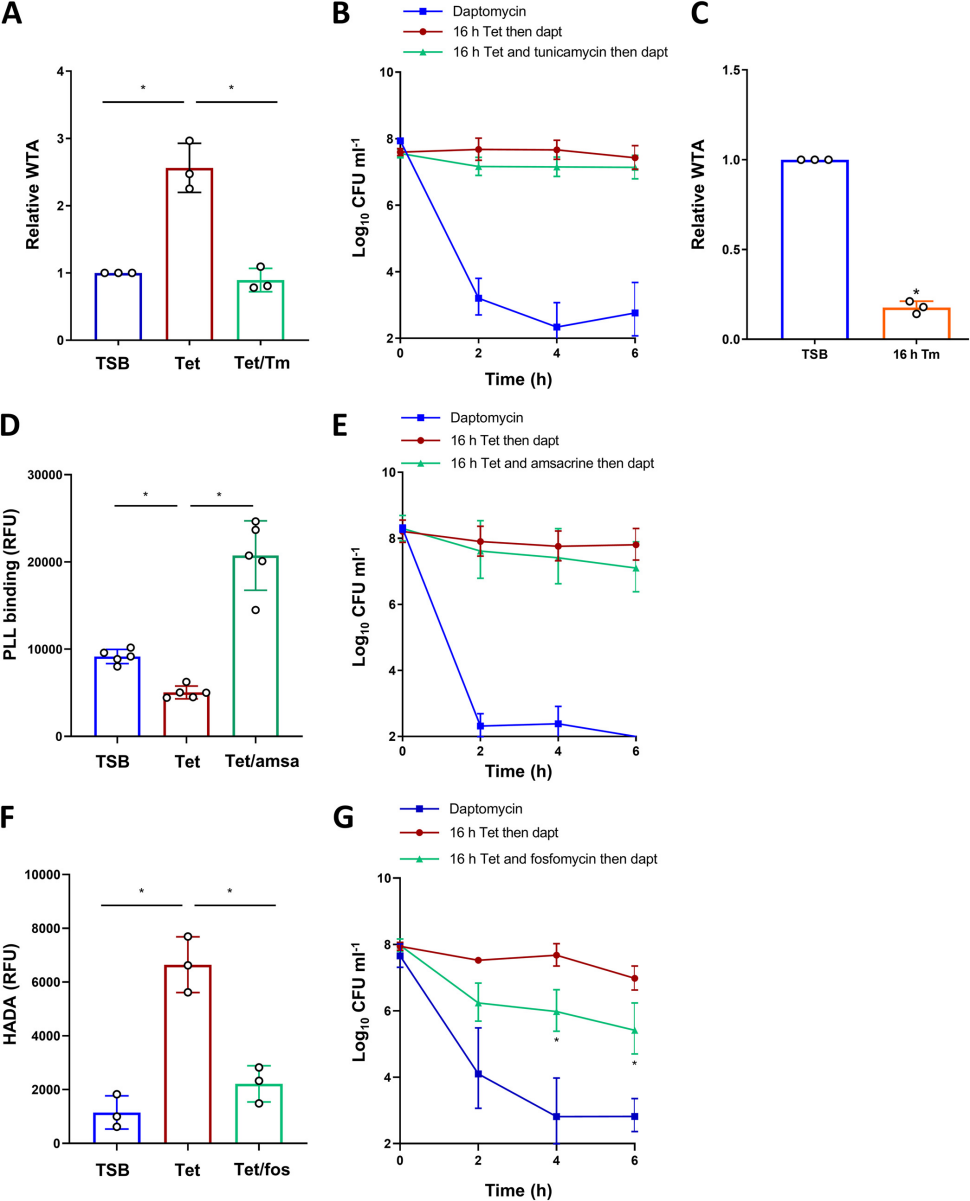
Daptomycin tolerance is due to the accumulation of peptidoglycan but not WTA. Exponential-phase S. aureus LAC* were growth-arrested with tetracycline ± 0.5 μg mL^−1^ tunicamycin for 16 h before (A) WTA was extracted, analyzed via PAGE, and quantified via Alcian blue staining or (B) the log_10_ CFU mL^−1^ were determined during a 6 h exposure to 20 μg mL^−1^ daptomycin. Exponential-phase S. aureus were growth-arrested with 0.5 μg mL^−1^ tunicamycin for 16 h, or not, before WTA was extracted, analyzed via PAGE, and quantified via Alcian blue staining (C). Exponential-phase S. aureus LAC* were growth-arrested with tetracycline ± 10 μg mL^−1^ amsacrine for 16 h before (D) the surface charge was measured using FITC-PLL or (E) the log_10_ CFU mL^−1^ were determined during a 6 h exposure to 20 μg mL^−1^ daptomycin. Exponential-phase S. aureus LAC* were growth-arrested with tetracycline ± 8 μg mL^−1^ fosfomycin for 16 h before (F) the peptidoglycan content was measured using HADA or (G) the log_10_ CFU mL^−1^ were determined during a 6 h exposure to 20 μg mL^−1^ daptomycin. The data in panels A, C, D, and F represent the mean ± the standard deviation of the indicated number of independent experiments. The data in panels B, E, and G represent the geometric mean ± the geometric standard deviation of three independent experiments. The data in panels A, D, and F were analyzed via a one-way ANOVA with Tukey’s *post hoc* test (*, *P* < 0.05). The data in panels B, E, and G were analyzed via a two-way ANOVA with Dunnett’s *post hoc* test (*, *P* < 0.05; tetracycline-arrested versus tetracycline + inhibitor-arrested). The data in panel C were analyzed via a Student's *t* test (*, *P* < 0.05) TSB, tryptic soy broth; Tet, tetracycline; Tm, tunicamycin; Amsa, amsacrine; Fos, fosfomycin; Dapt, daptomycin.

To do this, we growth-arrested bacteria with tetracycline in TSB in the presence of inhibitors of WTA synthesis, teichoic acid d-alanylation, or peptidoglycan synthesis and then measured the daptomycin tolerance. First, we showed that tunicamycin blocked the increase in WTA that occurred during the growth arrest with tetracycline, but the levels that were observed in the exponential-phase cells were maintained (Fig. 6A). Then, we measured the daptomycin susceptibility of the tetracycline plus tunicamycin-arrested bacteria. Despite having the same levels of WTA as did the exponential-phase bacteria, these cells had high levels of daptomycin tolerance, with no significant killing being observed by 6 h. Therefore, the accumulation of WTA that occurs during growth arrest was not required for daptomycin tolerance (Fig. 6B).

Since we had previously found that cells that were growth arrested with tunicamycin alone had a low level of tolerance (Fig. 2D), we examined the levels of WTA in these bacteria. This showed that cells that were growth arrested with tunicamycin alone had approximately 5-fold lower levels of WTA, relative to exponential-phase cells (Fig. 6C) or cells that were growth arrested with tetracycline plus tunicamycin (Fig. 6A). These data, together with those obtained from the WTA-deficient Δ*tagO* mutant (Fig. 4F), led us to conclude that a minimal amount of WTA is needed for tolerance but that the accumulation of the polymer above the levels observed in the exponential-phase cells was not required.

Next, we assessed the importance of the d-alanylation of teichoic acids for daptomycin tolerance using amsacrine, which inhibits DltB (52). We showed that this inhibitor worked under the conditions used by measuring the binding of fluorescently labeled poly-l-lysine (FITC-PLL), a positively charged polymer that is an indicator of bacterial surface charge (Fig. 6D). The exposure of bacteria that were treated with tetracycline and amsacrine to daptomycin revealed a high level of tolerance, demonstrating that the increased levels of teichoic acid d-alanylation seen in the growth arrested cells did not contribute to antibiotic survival (Fig. 6E), although the presence of some d-alanylation is required (Fig. 4H).

Finally, we investigated whether the growth arrest-induced accumulation of peptidoglycan mediated daptomycin tolerance. Peptidoglycan accumulation was inhibited using fosfomycin, which prevented the increase in HADA incorporation that was seen during the tetracycline-mediated growth arrest (Fig. 6F). In contrast to the tetracycline-arrested cultures, bacteria in which peptidoglycan accumulation had been prevented were significantly killed by daptomycin, with an over 2 log reduction in CFU counts being measured after a 6 h exposure to the lipopeptide antibiotic (Fig. 6G).

Therefore, while the presence of both WTA and its d-alanine modification are required for daptomycin tolerance (Fig. 4F, G, H), it is the accumulation of peptidoglycan, and not WTA, that is responsible for tolerance. The inhibition of peptidoglycan synthesis, for example, using fosfomycin, may therefore prevent the emergence of tolerance and thus may improve daptomycin treatment outcomes.

## DISCUSSION

In contrast to most bactericidal antibiotics, daptomycin has been reported to kill nonreplicating bacteria, including those that were growth-arrested via exposure to bacteriostatic drugs (10, 20, 21). However, previous work has shown that brief periods (<1 h) of antibiotic-mediated growth arrest reduced daptomycin-mediated killing, suggesting that the bactericidal activity of the lipopeptide antibiotic is affected by growth status. The aims of this work were to understand the degree to which growth arrest can reduce killing by daptomycin and to understand the mechanism responsible.

Several studies have shown that the antibiotic tolerance of nonreplicating bacteria occurs almost immediately once growth stops, and this may be partly due to a lack of metabolic activity and/or ATP (7–11, 15). In contrast, in this work, we show that growth arrest induces high levels of daptomycin tolerance via an active mechanism that requires time, nutrients, and peptidoglycan synthesis.

Tetracycline-mediated growth arrest led to an accumulation of major cell wall components, which agrees with previous work that shows that the inhibition of protein synthesis halts growth but not peptidoglycan biosynthesis in S. aureus (53). However, while the presence of WTA and d-alanylation were essential for tolerance, their accumulation was not. This is in concordance with previous work from our group that showed that peptidoglycan accumulation in bacteria incubated in serum was dependent upon the presence, but not the accumulation, of d-alanylated WTA (40). The increased levels of the d-alanylation of teichoic acids and WTA accumulation have been observed in some strains of daptomycin-resistant S. aureus (more commonly referred to as daptomycin nonsusceptible), although their role in antibiotic susceptibility was unclear (46, 47). Based on our findings, we hypothesize that d-alanylated WTA contributes to daptomycin resistance by enabling peptidoglycan accumulation (40). While this hypothesis would not explain all daptomycin resistance, it is supported by reports describing increased cell wall thickness in conjunction with increased levels of WTA and d-alanylation in some daptomycin nonsusceptible strains (54–58).

We confirmed that peptidoglycan accumulation was required for daptomycin tolerance via the partial digestion of this polymer with lysostaphin. This finding contrasts with the results of a previous study that showed that the complete removal of the cell wall led to high levels of daptomycin tolerance in S. aureus and E. faecalis (28). A potential reason for these differing findings is that our work only partially removed the cell walls from growth-arrested bacteria in growth media, whereas the previous study completely removed the cell walls from growing cells in hypertonic buffer (20% sucrose) (28). Furthermore, the complete removal of the cell wall may lead to lysis, even in hypertonic buffer, and, thereby, the release of membrane phosphatidylglycerol can sequester daptomycin and thereby severely reduce daptomycin activity (44, 45). Importantly, however, this previous work also showed that the inhibition of peptidoglycan synthesis using fosfomycin increased the antibacterial activity of daptomycin, and this result is in agreement with our work (28).

While there is clearly a key role for the cell wall in daptomycin tolerance that is caused by growth arrest, the inhibition of peptidoglycan did not completely restore susceptibility to the levels observed in replicating cells, indicating that other mechanisms may contribute. For example, membrane fluidity changes as cells enter the stationary-phase, and this alters daptomycin susceptibility (29). However, this previous work suggested that daptomycin binding to the membrane was unaltered, which contrasts with our observations here (29). As such, additional mechanisms by which nonreplicating cells have reduced daptomycin susceptibility remain to be determined.

We have previously found that human serum triggers daptomycin tolerance in S. aureus via two independent mechanisms: an increased abundance of peptidoglycan and changes to the membrane phospholipid composition (40). Although serum restricts staphylococcal growth, a key difference between daptomycin tolerance that is induced by growth arrest via antibiotics and that which occurs in serum is the absence of a role for changes to the membrane phospholipid composition, mediated by Cls2. While cardiolipin was required for serum-induced tolerance (40), we found no evidence that daptomycin tolerance required changes to the phospholipid composition. Furthermore, serum-induced tolerance required the presence of the antimicrobial peptide LL-37, and it was not triggered simply via the inhibition of S. aureus growth (40). Taken together, it appears that daptomycin tolerance can arise via distinct mechanisms in response to various conditions, but, in all cases, it appears to require the active remodeling of the cell envelope, rather than a lack of metabolic activity.

The reason why some antibiotics kill bacteria and others inhibit growth remains the subject of debate almost 80 years after penicillin was first used clinically (4–7). While there is currently no evidence to indicate that bactericidal antibiotics lead to better outcomes for patients than do bacteriostatic drugs (59–62), there is growing evidence that antibiotic tolerance is an underappreciated cause of treatment failure (13, 15, 63–65), and tolerance has also been shown to be a stepping stone to resistance (16, 17). A recent clinical example of this comes from a patient with relapsing MRSA bacteremia that was initially caused by a daptomycin-tolerant strain but gave rise to isolates that were daptomycin nonsusceptible during subsequent periods of relapse (66).

In addition to the potential clinical implications of antibiotic tolerance, it is anticipated that a better understanding of how antibiotics work and why certain combinations are synergistic may lead to more effective treatments. For example, the inhibition of daptomycin tolerance by fosfomycin or cephalexin suggests that they may have therapeutic value if used in combination with daptomycin. Indeed, several previous studies have shown synergy between daptomycin and fosfomycin or β-lactams *in vitro*, and our group showed that fosfomycin partially inhibited daptomycin tolerance that was induced by human serum (40, 67–69). There are also promising data from clinical studies, with the presence of a β-lactam or fosfomycin appearing to promote the clearance of S. aureus infections (63–65). However, combination therapy was generally more nephrotoxic than was daptomycin alone, and there was little evidence to suggest enhanced patient survival when daptomycin was used with a cell wall-targeting agent (30, 70, 71). Therefore, more work is needed to find the least toxic daptomycin combination therapy to extract clinical benefit (72).

In summary, we have shown that growth arrest results in tolerance to the antibiotic daptomycin, as occurs for many other bactericidal antibiotics. However, while growth arrest typically confers antibiotic tolerance quickly and without requiring metabolic activity, daptomycin tolerance arises in a time-dependent process via the active synthesis and accumulation of peptidoglycan.

## MATERIALS AND METHODS

### Bacterial strains and growth conditions.

The bacterial strains used in this study are shown in Table 1. Strains were routinely grown in tryptic soy broth (TSB; BD Biosciences) for 16 h at 37°C with shaking (180 rpm) or on tryptic soy agar (TSA; BD Biosciences). Where appropriate, media were supplemented with 10 μg mL^−1^ erythromycin.

**TABLE 1:** Strains used in this study

Strain	Characteristics^*a*^	Reference
USA300 LAC*	LAC strain of the USA300 CA-MRSA lineage, cured of LAC-p03 plasmid	74
USA300 LAC* Δ*tagO*	USA300 LAC* with the *tagO* gene deleted	75
USA300 LAC* Δ*ltaS*-S2	USA300 LAC*Δ*ltaS::erm* suppressor S2, Ery^r^	76
USA300 LAC* Δ*dltD*	USA300 LAC* with the *dltD* gene deleted, Ery^r^	40
USA300 LAC JE2	LAC strain of the USA300 CA-MRSA lineage cured of plasmids	77
USA300 LAC JE2 *mprF*::Tn	USA300 LAC JE2 with a *bursa aurealis* transposon insertion in *mprF*, Ery^r^	77
USA300 LAC JE2 *cls1*::Tn	USA300 LAC JE2 with a *bursa aurealis* transposon insertion in *cls1*, Ery^r^	77
USA300 LAC JE2 *cls2*::Tn	USA300 LAC JE2 with a *bursa aurealis* transposon insertion in *cls2*, Ery^r^	77
USA300 LAC JE2 *crtM*::Tn	USA300 LAC JE2 with a *bursa aurealis* transposon insertion in *crtM*, Ery^r^	77

a Ery, erythromycin.

### Generation of mid-exponential-phase and growth-arrested cultures.

The mid-exponential-phase cultures were generated via the dilution of overnight cultures to 10^7^ CFU mL^−1^, and this was followed by growth for 2 h at 37°C with shaking (180 rpm) until 10^8^ CFU mL^−1^ were reached. To generate the growth-arrested cultures, bacteriostatic concentrations of the appropriate antibiotic were added (Table 2). Due to the different susceptibilities of some mutants to some antibiotics, the concentrations that were used were optimized for each mutant. Except where stated, growth arrest was performed in TSB for 16 h at 37°C with shaking (180 rpm). As bacteriostatic concentrations were used, the CFU mL^−1^ remained constant (10^8^ CFU mL^−1^). Where appropriate, growth arrest was performed in phosphate-buffered saline (PBS), cation-adjusted Müller Hinton broth (MHB), or MHB supplemented with 2.5 g L^−1^ (13.9 mM) glucose or equimolar concentrations of galactose, sorbitol, sucrose, raffinose, or lactose. For most of the experiments, the cultures were incubated at 37°C with shaking (180 rpm), but some assays were done at 4°C or without shaking.

**TABLE 2:** Concentrations of antibiotics used in this study

Antibiotic^*a*^	Supplier	Strain	Concentration (μg mL^−1^)
Tetracycline (TSB)	Sigma	LAC* WT	1.25
		LAC* Δ*tagO*	0.156
		LAC* Δ*ltaS*-S2	0.078
		LAC* Δ*dltD*	0.3125
		JE2 WT	0.625
		JE2 *mprF*::Tn	0.625
		JE2 *cls1*::Tn	0.625
		JE2 *cls2*::Tn	0.625
		JE2 *crtM*::Tn	0.625
Tetracycline (MHB)	Sigma	LAC* WT	5
Chloramphenicol	Sigma	LAC* WT	20
Ciprofloxacin	Sigma	LAC* WT	160
Cephalexin	Tokyo Chemical Industry	LAC* WT	32
Tunicamycin	Abcam	LAC* WT	0.5
AFN−1252	MedChemExpress	LAC* WT	0.15
Fosfomycin	Tokyo Chemical Industry	LAC* WT	8
Amsacrine	Sigma	LAC* WT	10
Lysostaphin	Sigma	LAC* WT	1

a TSB, tryptic soy broth; MHB, Müller Hinton broth.

### Daptomycin killing assays.

The mid-exponential phase and growth-arrested cultures (3 mL) were generated as described above. CaCl_2_ was added to a final concentration of 1.25 mM, and daptomycin was added to 20 μg mL^−1^. The cultures were incubated at 37°C with shaking (180 rpm) for 6 h. At each time point, aliquots were taken, serially diluted 10-fold in PBS, and plated onto TSB to determine the CFU mL^−1^. Where appropriate, after growth arrest, cells were incubated with 1 μg mL^−1^ lysostaphin in TSB for 1 h at 37°C to partially remove the cell wall prior to the addition of daptomycin.

### Measurements of daptomycin binding.

Daptomycin was labeled with the BoDipy fluorophore as described previously (40). Cultures (3 mL) of exponential-phase or growth-arrested bacteria, with 10^8^ CFU mL^−1^ in each at the beginning of the assay, were incubated at 37°C with shaking (180 rpm) with 80 μg mL^−1^ BoDipy-daptomycin in TSB supplemented with 1.25 mM CaCl_2_. This concentration of antibiotic was used because BoDipy-daptomycin is approximately fourfold less active than is native daptomycin (73). As such, 80 μg mL^−1^ BoDipy-daptomycin is equivalent to the 20 μg mL^−1^ daptomycin concentration that was used in the other assays described in the manuscript. Every 2 h, aliquots were taken and washed in PBS three times. Samples (200 μL) were put into black-walled, flat-bottomed 96-well plates, and the fluorescence was measured using a TECAN Infinite 200 PRO microplate reader (excitation 490 nm; emission 525 nm), These values were divided by the OD_600_ measurements to normalize for differences in cell density. Alternatively, daptomycin binding was investigated via fluorescence microscopy as described below.

### Measurements of membrane depolarization.

Membrane polarity was measured using 3,3′-dipropylthiadicarbocyanine iodide (DiSC_3_(5); Thermo Fisher Scientific) as described previously (40). This dye binds to polarized membranes, which quenches its fluorescence and results in higher fluorescence values correlating with increased membrane depolarization. Exponential-phase and growth-arrested cultures were exposed to 20 μg mL^−1^ daptomycin for 6 h at 37°C in 3 mL TSB supplemented with 1.25 mM CaCl_2_. As for the daptomycin binding assays, each culture contained 10^8^ CFU mL^−1^ at the beginning of the assay. Every 2 h, 200 μL aliquots were taken into black-walled, flat-bottomed 96-well plates. DiSC_3_(5) was added to a final concentration of 1 μM and mixed. The plate was then incubated statically at 37°C for 5 min. Fluorescence was measured using a TECAN Infinite 200 PRO microplate reader (excitation 622 nm; emission 670 nm), and the values were divided by the OD_600_ measurements to normalize for differences in cell density.

As a positive control, bacteria were depolarized using the potassium ionophore nigericin (500 μg mL^−1^) for 1 h before the addition of DiSC_3_(5), and the fluorescence was measured as described above.

### Measurements of membrane permeability.

Membrane permeability was measured using propidium iodide (PI; Sigma), a membrane-impermeant dye which fluoresces when bound to DNA, as described previously (40). Exponential-phase and growth-arrested cultures (10^8^ CFU mL^−1^ at the start of the assay) were exposed to 20 μg mL^−1^ daptomycin for 6 h at 37°C in 3 mL TSB supplemented with 1.25 mM CaCl_2_. Every 2 h, 200 μL aliquots were washed three times with PBS and added into a black-walled, flat-bottomed 96-well plate, and PI was added to a final concentration of 2.5 μM. Fluorescence was measured using a TECAN Infinite 200 PRO microplate reader (excitation 535 nm; emission 617 nm), and the values were divided by the OD_600_ measurements to normalize for differences in cell density.

### Phase contrast and fluorescence microscopy.

Exponential-phase or growth-arrested cultures were incubated with BoDipy-daptomycin as described above. After 2 h, aliquots were taken, washed three times in PBS, and fixed in 4% paraformaldehyde. To measure peptidoglycan synthesis, exponential-phase and growth-arrested cultures were generated with the addition of 25 μM HADA. The samples were washed three times in PBS and fixed in 4% paraformaldehyde.

Aliquots of fixed bacteria (2 μL) were spotted onto agarose (1.2% in water) on microscope slides and covered with a cover slip. Images were taken with a Zeiss Axio Imager.A1 microscope that was coupled to an AxioCam MRm and a 100× objective. BoDipy-daptomycin was detected using a green fluorescent protein filter set, and HADA was detected using a DAPI filter set. The images were processed using the Zen 2012 software package (blue edition). Within an experiment, the microscopy of all samples was performed at the same time and using identical settings so as to enable comparisons to be made between samples.

### Extraction and quantification of WTA.

WTA was extracted from 40 mL cultures of exponential-phase or growth-arrested cultures as described previously (40). Briefly, the cultures were washed with 50 mM MES, pH 6.5, and incubated at 100°C for 1 h in 4% SDS and 50 mM MES, pH 6.5. The samples were then washed twice in 4% SDS and 50 mM MES, once with 2% NaCl and 50 mM MES, and once with 50 mM MES before being resuspended in 1 mL 20 mM Tris-HCl (pH 8), 0.5% SDS, and 20 μg mL^−1^ proteinase K. The samples were then incubated for 4 h at 50°C (shaking at 1,400 rpm). The pellet was recovered, washed with 2% NaCl and 50 mM MES, and washed three times with water before being resuspended in 1 mL 0.1 M NaOH and incubated at 20°C for 16 h (shaking at 1,400 rpm). After centrifugation at 16,000 × *g* for 1 min, the supernatant was neutralized with 250 μL 1 M Tris-HCl, pH 7.8, and stored at −20°C. The CFU counts from each culture were measured as described above to ensure that the extracts represented equal numbers of bacterial cells for all conditions tested (10^8^ CFU mL^−1^).

Purified WTA was loaded on 20% native polyacrylamide gels and run at 120 V in Tris-Tricine running buffer (0.1 M Tris, 0.1 M Tricine) before being stained with 0.1% Alcian blue in 3% acetic acid. The gels were destained with water and imaged using a Gel Doc EZ Imager (Bio-Rad). The WTA intensity was quantified using ImageJ.

### Extraction, detection, and quantification of LTA.

Exponential-phase and growth-arrested cultures (10 mL) were resuspended in 1 mL PBS and transferred to screw cap tubes containing approximately 100 μL 0.1 mm glass beads. The cells were lysed at room temperature using a FastPrep-24 (MP Biomedicals) machine (4 cycles of 6.5 m/s for 40 s, and this was followed by a 1 min rest). The tubes were centrifuged at 200 × *g* for 1 min to settle the beads, and 500 μL of the supernatant was removed into a new tube. Centrifugation (16,000 × *g* for 15 min) pelleted the cellular debris, including the LTA. The supernatant was discarded, and the pellet was resuspended in 100 μL 2× Laemmli sample buffer (4% SDS, 20% glycerol, 10% β-mercaptoethanol, 0.02% bromophenol blue, 0.125 M Tris-HCl, pH 6.8). The samples were incubated at 95°C for 20 min and were then centrifuged at 17,000 × *g* for 5 min before the supernatant, which contained LTA, was moved to a fresh Eppendorf and was stored at −20°C.

As described for WTA extractions, CFU counts from each culture were measured as described above and adjusted to ensure that the extracts represented equal numbers of bacterial cells for all conditions tested.

LTA extracts (10 μL) were run on 15% polyacrylamide gels and transferred to PVDF membranes using a Trans-Blot Turbo transfer system (Bio-Rad). After blocking with 5% milk and 1% BSA in TBST, LTA was detected with an anti-LTA primary antibody (MAb 55; HycultBiotech; 1:5000 dilution) and an HRP-goat anti-mouse IgG secondary antibody (Abcam; 1:10,000 dilution). The LTA was visualized using the Amersham ECL Prime Western blotting detection reagent (GE Healthcare) and a ChemiDoc imaging system (Bio-Rad). The LTA intensity was quantified using ImageJ.

### Measurement of surface charge.

The bacterial surface charge was determined using fluorescein isothiocyanate-labeled poly-l-lysine (FITC-PLL). Aliquots (1 mL) of exponential-phase and growth-arrested cultures were incubated with 80 μg mL^−1^ FITC-PLL for 10 min at room temperature in the dark before being washed three times in PBS. 200 μL was then moved to a black-walled 96-well plate, and the fluorescence was measured using a TECAN Infinite PRO 200 plate reader (excitation 485 nm; emission 525 nm).

### Measurements of HADA incorporation into peptidoglycan using a plate reader.

Peptidoglycan synthesis was measured using HCC-amino-d-alanine (HADA), a fluorescent d-amino acid analogue. Exponential-phase and growth-arrested cultures were generated with the addition of 25 μM HADA. The samples were washed three times in PBS, and 200 μL aliquots were moved to black-walled 96-well plates. The fluorescence was measured using a TECAN Infinite 200 PRO microplate reader (excitation 405 nm; emission 450 nm).

### Measurements of HADA incorporation into peptidoglycan via flow cytometry.

Exponential-phase and growth-arrested cultures were generated with the addition of 25 μM HADA. The samples were washed three times in PBS and fixed in 4% paraformaldehyde. The samples were analyzed using an Amnis CellStream system. The median fluorescence value from 10,000 cells per sample was determined for three independent replicates.

### Determination of MICs.

Doubling dilutions of daptomycin were prepared in MHB supplemented with 1.25 mM CaCl_2_. Wells were inoculated with 5× 10^5^ CFU mL^−1^ bacteria and incubated statically at 37°C for 17 h. The MIC was determined as the lowest concentration of antibiotic with no visible growth.

### Statistical analyses.

The CFU data were log_10_ transformed. The data were analyzed via a Student’s *t* test, one-way analysis of variance (ANOVA), or two-way ANOVA with a *post hoc* test to correct for multiple comparisons, as is described in the figure legends, using GraphPad Prism (v8.0).
