# Supplementary figures and images for: Growth Arrest of Staphylococcus aureus Induces Daptomycin Tolerance via Cell Wall Remodelling
Source: mBio. 2023 Feb 1;14(1):e03558-22. doi: 10.1128/mbio.03558-22 (PMC9973334; doi:10.1128/mbio.03558-22)

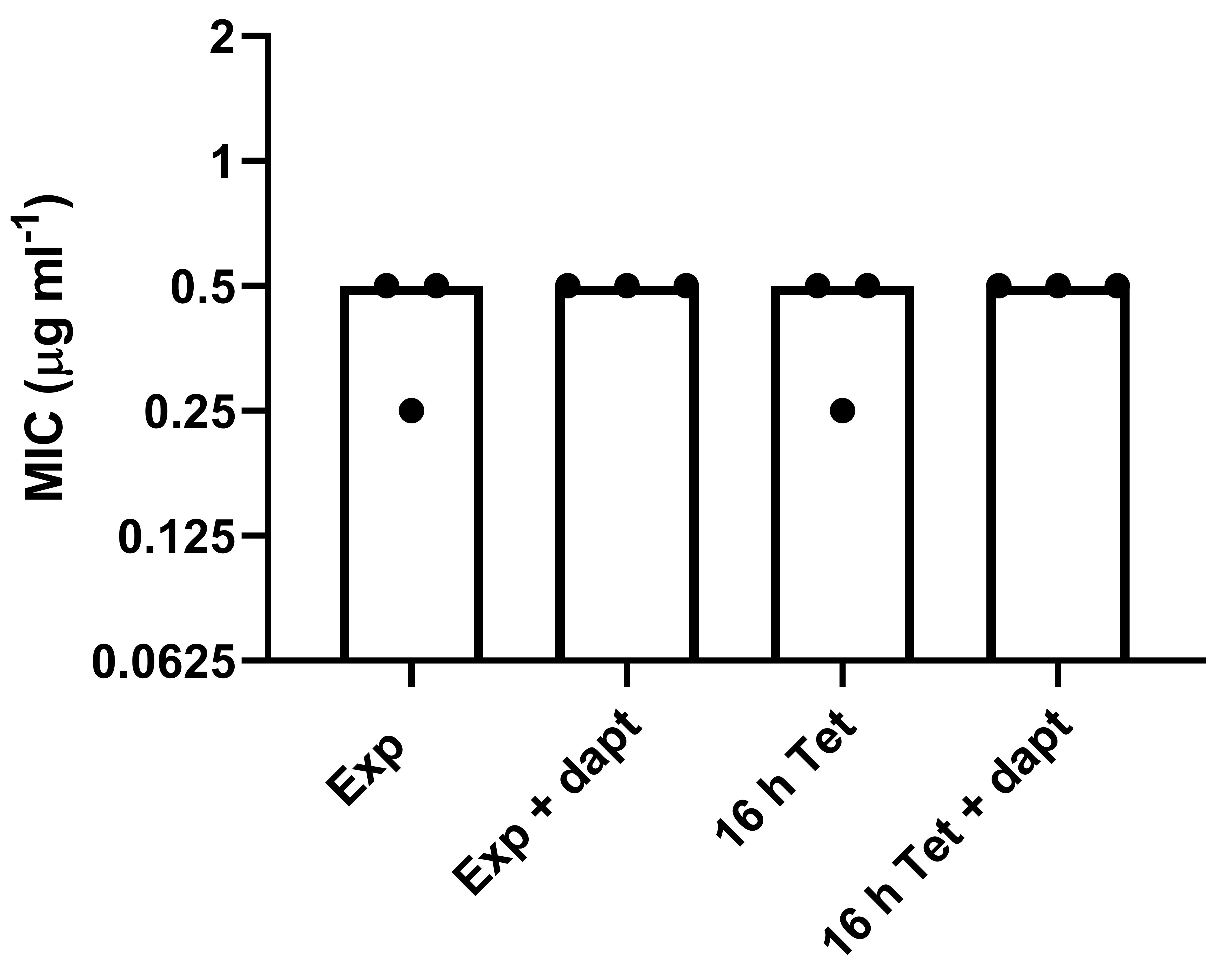

Supplement: FIG S1 [file mbio.03558-22-s0001.tif]

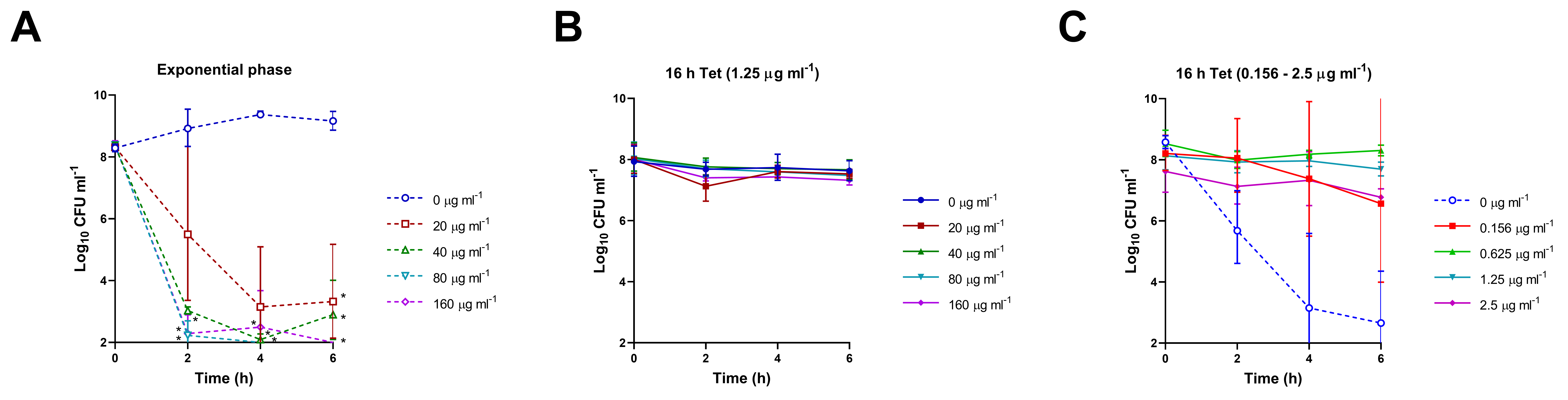

Supplement: FIG S2 [file mbio.03558-22-s0002.tif]

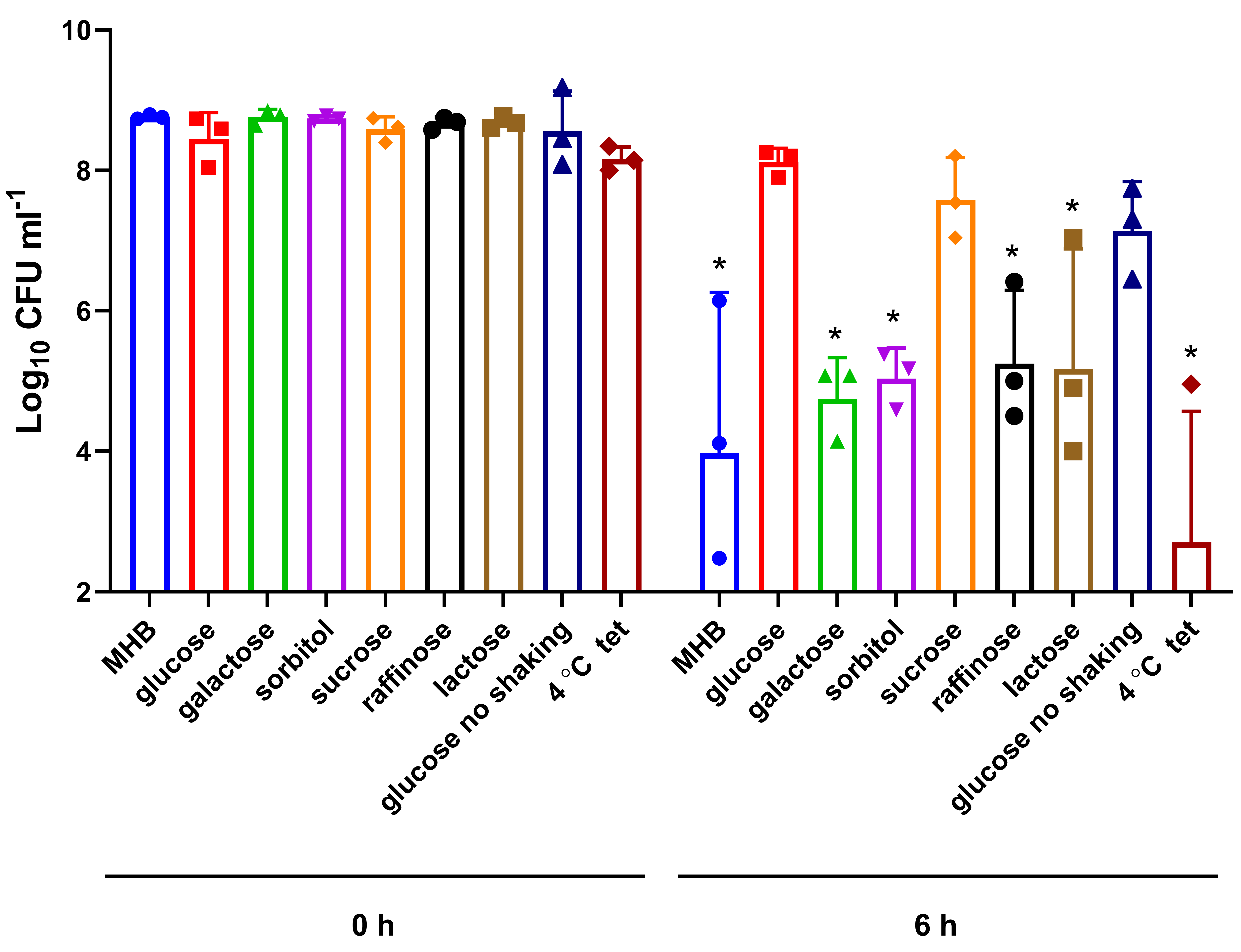

Supplement: FIG S3 [file mbio.03558-22-s0003.tif]

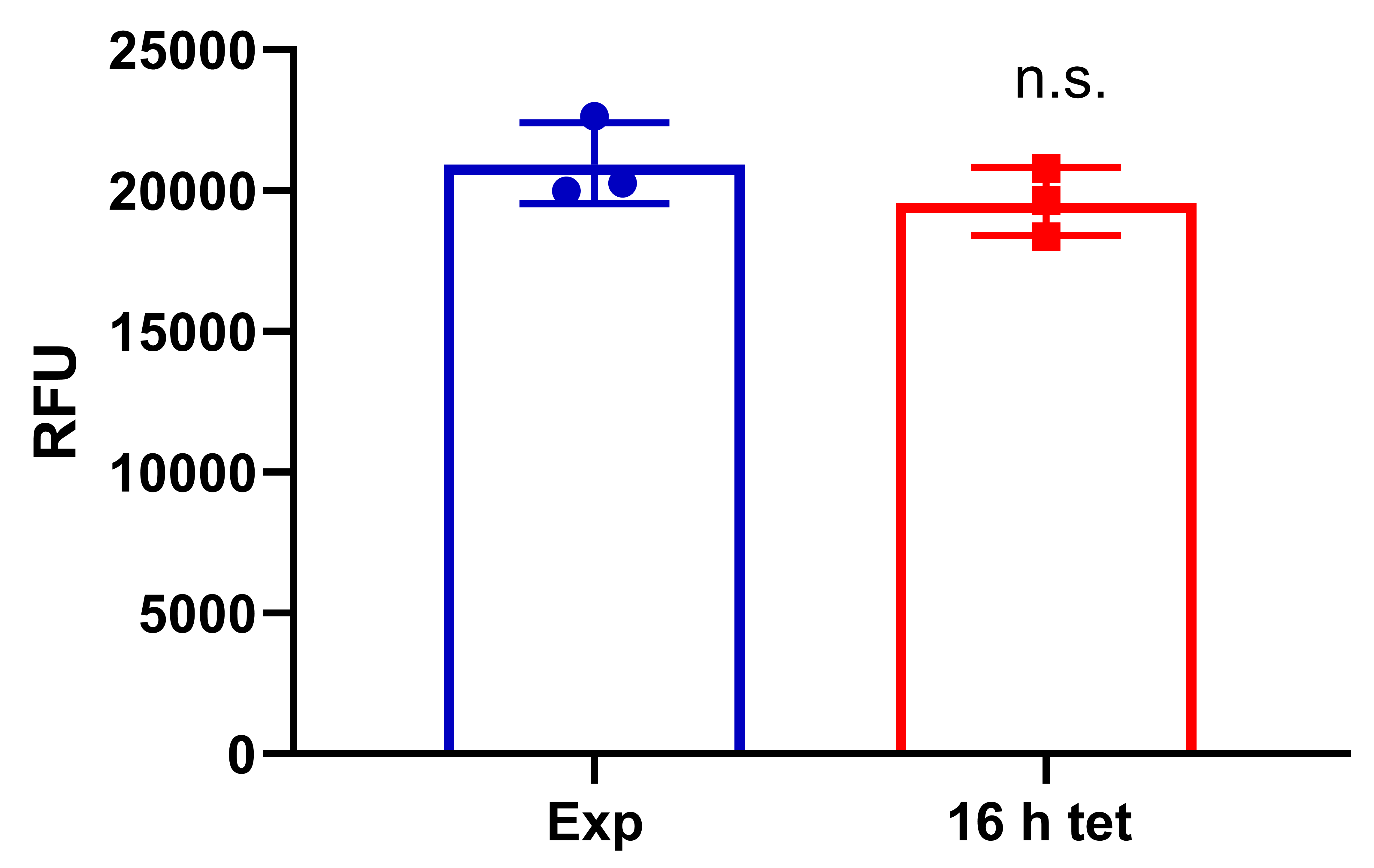

Supplement: FIG S4 [file mbio.03558-22-s0004.tif]
